# A viable hypomorphic *Arnt2* mutation causes hyperphagic obesity, diabetes and hepatic steatosis

**DOI:** 10.1242/dmm.035451

**Published:** 2018-12-18

**Authors:** Emre E. Turer, Miguel San Miguel, Kuan-wen Wang, William McAlpine, Feiya Ou, Xiaohong Li, Miao Tang, Zhao Zang, Jianhui Wang, Braden Hayse, Bret Evers, Xiaoming Zhan, Jamie Russell, Bruce Beutler

**Affiliations:** 1 Center for the Genetics of Host Defense, University of Texas Southwestern Medical Center, Dallas, TX, 75390-8505, USA; 2 Department of Internal Medicine, Division of Gastroenterology, University of Texas Southwestern Medical Center, Dallas, TX, 75390-8505 USA; 3 Department of Pathology, University of Texas Southwestern Medical Center, Dallas, TX, 75390-8505 USA

**Keywords:** *N*-ethyl-*N*-nitrosourea, ENU, Obesity, Hyperphagia

## Abstract

Aryl hydrocarbon receptor nuclear translocator 2 (ARNT2) is a member of the basic helix-loop-helix/PER-ARNT-SIM (bHLH/PAS) transcription factor family. ARNT2 heterodimerizes with several members of the family, including single-minded homolog-1 (SIM1) and neuronal PAS domain protein 4 (NPAS4), primarily in neurons of the central nervous system. We screened 64,424 third-generation germline mutant mice derived from *N*-ethyl-*N*-nitrosourea (ENU)-mutagenized great-grandsires for weight abnormalities. Among 17 elevated body weight phenotypes identified and mapped, one strongly correlated with an induced missense mutation in *Arnt2* using a semidominant model of inheritance. Causation was confirmed by CRISPR/Cas9 gene targeting to recapitulate the original ENU allele, specifying Arg74Cys (R74C). The CRISPR/Cas9-targeted (*Arnt2*^R74C/R74C^) mice demonstrated hyperphagia and increased adiposity as well as hepatic steatosis and abnormalities in glucose homeostasis. The mutant ARNT2 protein showed decreased transcriptional activity when coexpressed with SIM1. These findings establish a requirement for ARNT2-dependent genes in the maintenance of the homeostatic feeding response, necessary for prevention of obesity and obesity-related diseases.

## Abstract (precis)

**Summary:** A forward genetic approach identified genes involved in maintaining normal body weight. A mutation in *Arnt2* caused obesity by regulating food intake.

## INTRODUCTION

Obesity is growing in prevalence, with more than a third of the world population currently overweight or obese. Comorbid conditions, such as type 2 diabetes, coronary artery disease, stroke and liver disease, cause a large economic health burden in the United States. Human genetic studies have largely demonstrated a polygenic etiology for these diseases with little insight into individual gene function. Rare familial monogenic obesity syndromes have been informative in elucidating underpinnings of obesity, demonstrating roles for genes such as *SIM1* and *TUB* in obesity (Barsh et al., 2000; van der Klaauw and Farooqi, 2015). These monogenic disorders account for a minute fraction of human obesity, which emphasizes the importance of finding new contributory loci (Hendricks et al., 2017; Kleinert et al., 2018).

The essential molecular components required for maintenance of body weight can be revealed by random germline mutagenesis, particularly when combined with automated mapping (Wang et al., 2015). Accordingly, we carried out a forward genetic screen of mice with *N*-ethyl-*N*-nitrosourea (ENU)-induced mutations, assessing for deviation from normal body weight. Here, we describe mice bearing a mutation in *Arnt2* (R74C), which developed hyperphagic obesity as well as fatty liver and glucose intolerance. A member of the basic helix-loop-helix/PER-ARNT-SIM (bHLH/PAS) transcription factor family, *Arnt2* encodes the aryl hydrocarbon receptor nuclear translocator 2 (ARNT2), which is expressed in the CNS and is necessary for production of secretory hormones in the hypothalamus (Hankinson, 2008; Hosoya et al., 2001; Keith et al., 2001). In marked contrast to mice with null mutations of *Arnt2*, homozygous *Arnt2*^R74C^ mice are born at expected Mendelian frequencies and survive to adulthood. Our findings reveal an important role for ARNT2 and ARNT2-dependent genes in regulating feeding behavior and body fat mass, advancing our understanding of the mechanisms controlling these traits.

## RESULTS

### Weight-based screening of ENU-induced mutant mice

**Fig. 1.**
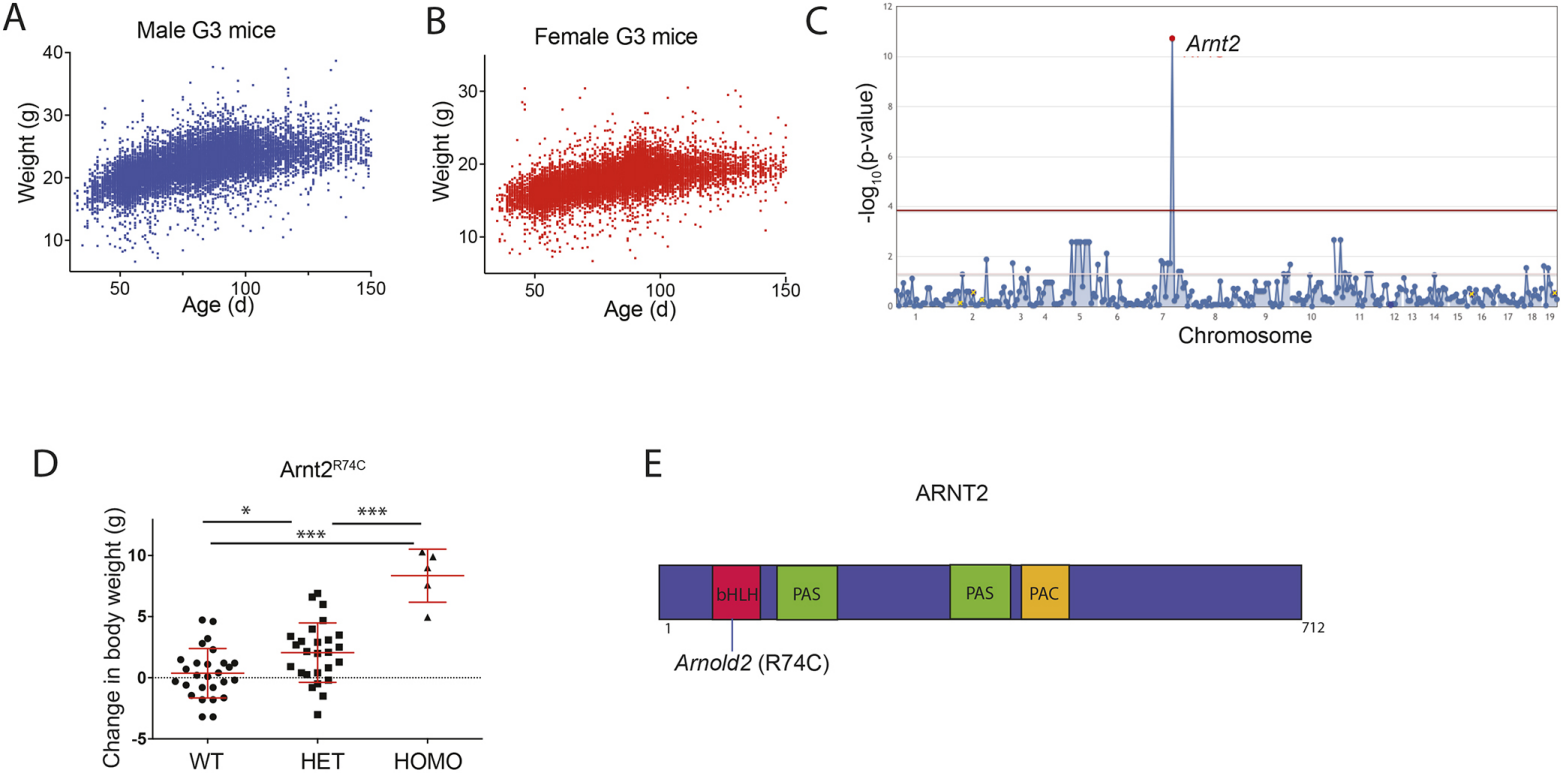
**Increased body weight associated with a mutation in *Arnt2*.** (A,B) Body weights of 14,394 male (A) and 14,956 female (B) G3 mice. (C) The Manhattan plot shows –log_10_(*P*-value) (*y*-axis) plotted against the chromosome positions of all mutations (*x*-axis) identified in the G1 male founders of four pedigrees containing the *Arnt2*^R74C^ mutation. Horizontal pink and red lines represent thresholds of *P*=0.05, without and with Bonferroni correction, respectively. (D) Body weight change relative to the average body weight of age- and sex-matched wild-type C57BL/6J mice. Homozygous *Arnt2* mutant (HOMO; *n*=5), heterozygous *Arnt2* mutant (HET; *n*=26) and *Arnt2^+/+^* (WT; *n*=27) mice. Data points represent individual mice. Mean and s.d. are indicated. *P<0.05 and ***P<0.0005 for differences between marked genotype by one-way ANOVA with *post-hoc* Tukey's multiple comparison test. (E) Diagram of the protein domains of mouse ARNT2. The location of the *Arnold2* mutation is shown. bHLH, basic helix-loop-helix; PAS, PER/ARNT/SIM domain; PAC, motif C-terminal to PAS motifs.

To identify genes required for the maintenance of normal body weight, we screened C57BL/6J third-generation (G3) mice bred to carry homozygous and heterozygous mutations induced by ENU in their great-grandsires (G0 mice). G3 mice were genotyped (prior to phenotypic screening) for all mutations present in their pedigree, passed down from a single G1 grandsire that was subjected to exome sequencing. G3 mice were individually weighed after weaning, and their ages noted (Fig. S1). Because G3 mice from a given pedigree are born over a period of 2-3 months and phenotypically screened as a group, raw body weight data could not be used directly for linkage analysis. Instead, we determined the body weight change of an individual mouse relative to the average body weight of age- and sex-matched mice among a group of 10,807 C57BL/6J G3 mice; these mice were housed under the same conditions and carried ENU-induced mutations. Body weight change was used as a continuous variable for linkage analysis. A total of 64,424 G3 mice from 2356 pedigrees were screened for phenovariance in the body weight screen (Fig. 1A,B). These pedigrees were a repository of 98,684 mutations in 17,702 genes that were transmitted to homozygosity three or more times and examined for phenotypic effects. Among the 98,684 mutations, 5906 mutations in 3856 genes were putative null alleles (premature stop codons or critical splice junction errors), while 29,693 mutations in 7789 genes were predicted to be ‘probably damaging’ by PolyPhen-2 (Polymorphism Phenotyping v2) with a score of 0.95 or greater (Adzhubei et al., 2010) (Table 1). In the body weight screen to date, we estimate that 26.5% of the genome has been modified with truly damaging mutations that have been tested in the homozygous state at least three times (Wang et al., 2018).

**Table 1.**
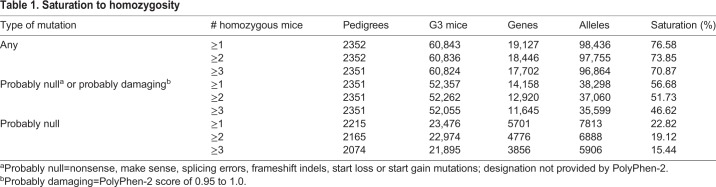
**Saturation to homozygosity**

During the course of the body weight screen, linkage mapping identified putative causative mutations in 17 pedigrees (Table 2). For each mutation, there were at least two homozygous variant mice and their average weight was at least 3 g more than that of wild-type or heterozygous controls, resulting in *P*-values for linkage of less than 5×10^−4^ with Bonferroni correction. Several mutations in genes known to cause increased body weight in mice were identified, including mutations in *Alms1*, *Clock*, *Ksr2*, *Socs2*, *Tub*, *Lep*, *Lepr* and *Mc4r*, validating the utility of the screen. Mutations in human *KSR2*, *MC4R*, *LEP* and *LEPR* have been described as monogenic causes of obesity (Barsh et al., 2000; Turcot et al., 2018), confirming that this screen has relevance to human monogenic obesity disorders.

**Table 2.**
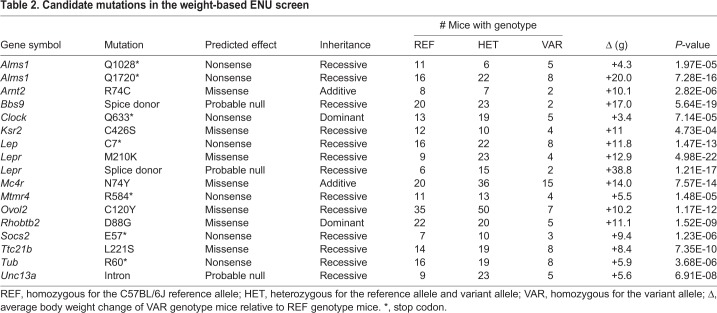
**Candidate mutations in the weight-based ENU screen**

### A semidominant mutation in *Arnt2* is associated with increased body weight

One *Arnt2* missense allele was detected in five pedigrees sharing a common G0 ancestor and was found in homozygous state in three of these pedigrees. The data from all five pedigrees were pooled into a position-specific superpedigree (Wang et al., 2015) (Fig. 1C), revealing a highly significant semidominant effect on body weight (*P*=1.828×10^−11^). Homozygosity for the allele, named *Arnold2*, was associated with an average increase of 7.9 g in age-/sex-adjusted body weight over wild-type littermates across the five pedigrees (Fig. 1D). The *Arnold2* mutation results in an arginine to cysteine substitution at amino acid 74 (R74C) in the ARNT2 protein; this change was predicted ‘probably damaging’ by PolyPhen-2 (score 1.0). Arg74 is located in the basic helix-loop-helix domain of ARNT2 (Fig. 1E). These data implicate the R74C mutation in *Arnt2* as causative for the obesity phenotype in the *Arnold2* mice.

**Fig. 2.**
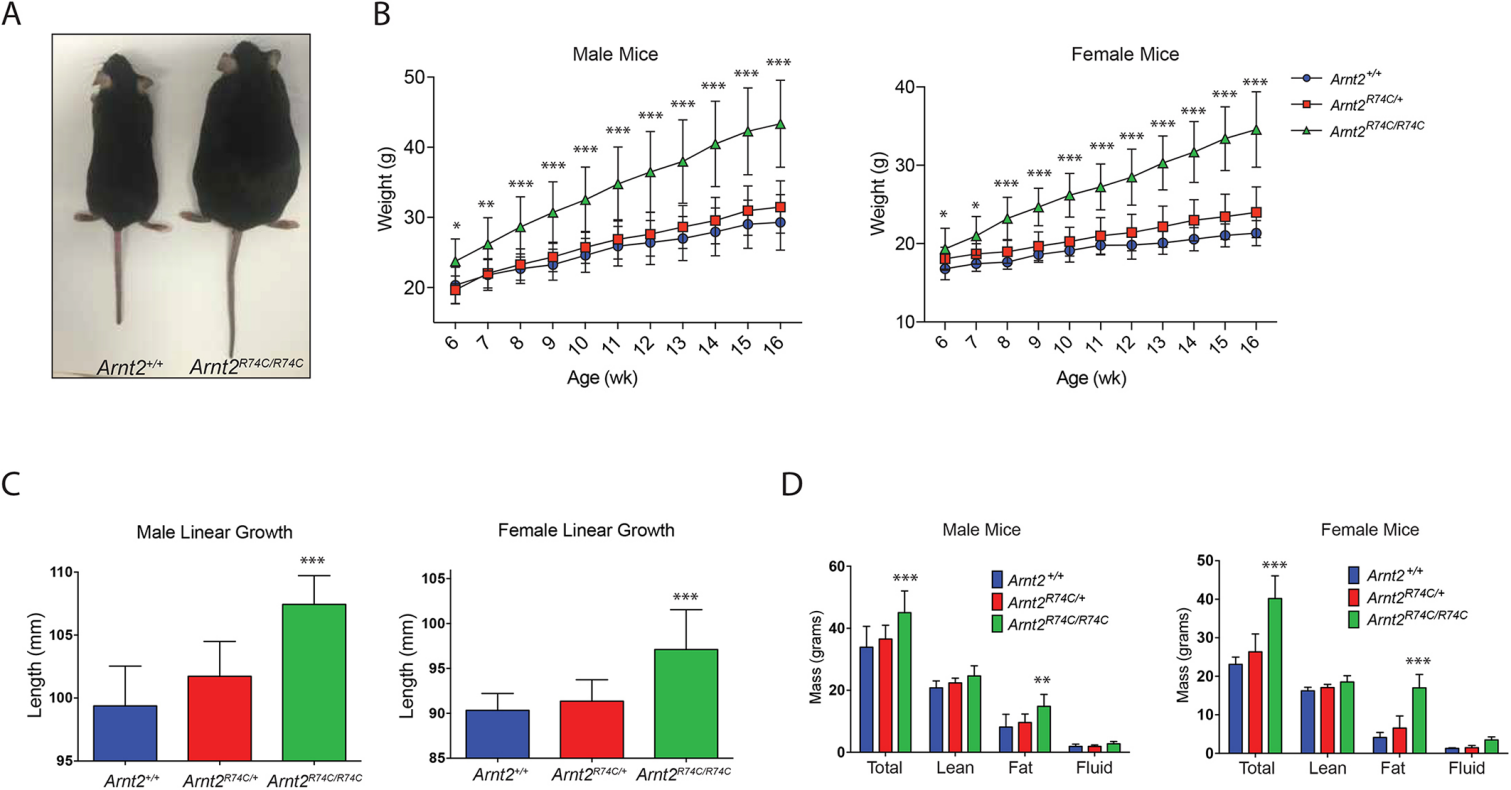
**Validation of the *Arnt2*^R74C^ mutation as a monogenic cause of obesity.** (A) Representative photograph of 6-month-old female *Arnt2^+/+^* and *Arnt2*^R74C/R74C^ littermates. (B) Growth curves of male (*n*=14, 25 and 16 for *Arnt2*^+/+^, *Arnt2*^R74C/+^ and *Arnt2*^R74C/R74C^, respectively) and female (*n*=13, 22 and 12 for *Arnt2*^+/+^, *Arnt2*^R74C/+^ and *Arnt2*^R74C/R74C^, respectively) mice from 6 weeks of age to 16 weeks of age. (C) Length of male (*n*=8, 11 and 16 for *Arnt2*^+/+^, *Arnt2*^R74C/+^ and *Arnt2*^R74C/R74C^, respectively) and female (*n*=9, 20 and 9 for *Arnt2^+/+^*, *Arnt2*^R74C/+^ and *Arnt2*^R74C/R74C^, respectively) mice at 4-5 months of age. (D) Magnetic resonance spectroscopy body composition analysis for male (*n*=5, 9 and 11 for *Arnt2^+/+^*, *Arnt2*^R74C/+^ and *Arnt2*^R74C/R74C^, respectively) and female (*n*=8, 12 and 5 for *Arnt2^+/+^*, *Arnt2*^R74C/+^ and *Arnt2*^R74C/R74C^, respectively) mice at 4-5 months of age. Data are a composite of all mice measured. *P<0.05, **P<0.005 and ***P<0.0005 for differences between marked genotype and *Arnt2*^+/+^ or *Arnt2*^R74C/+^ (at a single time point in B) by one-way ANOVA with *post-hoc* Tukey's comparison test.

In order to verify causation, CRISPR/Cas9-mediated gene targeting was used to generate a single-nucleotide replacement allele (C→T at chr7_84,347,530), causing the same amino acid change as that caused by the ENU-induced mutation (R74C) (Fig. S2). Like homozygous *Arnold2* mice, *Arnt2*^R74C/R74C^ mice were born at normal Mendelian frequencies and survived to adulthood (99 *Arnt2*^R74C/R74C^ of 399 total offspring from heterozygous parents; 24.8%). The *Arnt2*^R74C/R74C^ mice showed increased body weights compared to wild-type controls (Fig. 2A). Growth curves of *Arnt2*^R74C/R74C^ male and female mice showed increased body weights for all time points examined from 6 weeks to 16 weeks of age as compared to *Arnt2*^R74C/+^ and *Arnt2*^+/+^ mice (Fig. 2B). By 4 months of age, *Arnt2*^R74C/R74C^ male and female mice weighed on average 14.1 and 13.1 g, respectively, more than age-/sex-matched *Arnt2*^+/+^ littermates (Fig. 2B). The average linear growth of male and female animals was increased 8.1 mm and 6.8 mm over control littermates, respectively (Fig. 2C).

In order to examine the overall body composition of the *Arnt2*^R74C/R74C^ mice, magnetic resonance spectroscopy (MRS) was employed. At 4 months of age, the *Arnt2*^R74C/R74C^ mice showed increased absolute adiposity amounts compared to wild-type and heterozygous littermates; the phenotype was especially prominent in female mice (Fig. 2D). Taken together, the findings from the *Arnt2*^R74C/R74C^ mice confirmed that the *Arnold2* mutation caused increased body weight, and is a monogenic cause of obesity in mice.

### Diabetes and hepatic steatosis in *Arnt2*^R74C/R74C^ mice

**Fig. 3.**
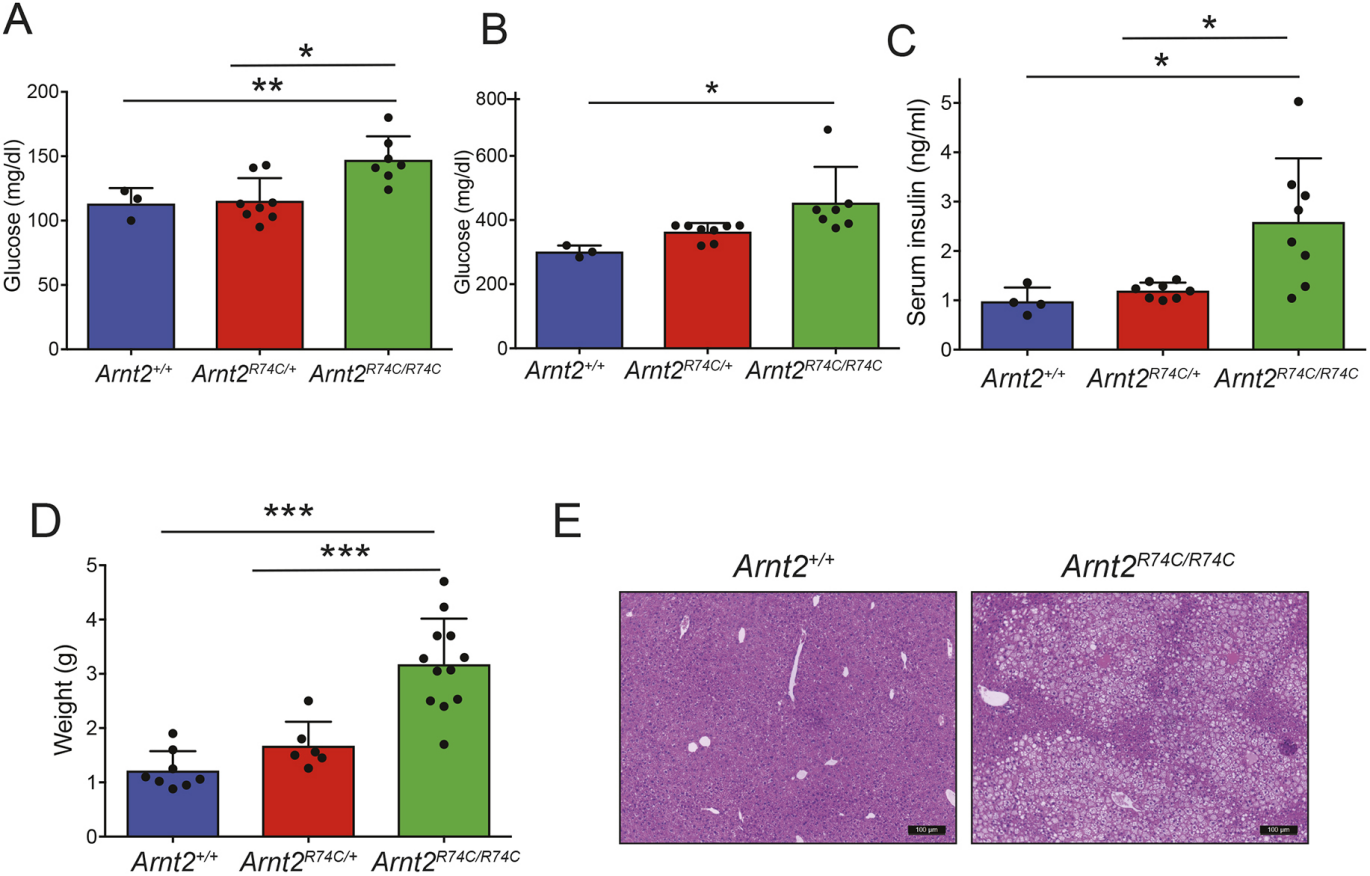
**Dysregulated glucose homeostasis and hepatic steatosis in *Arnt2*^R74C/R74C^ mice.** (A,B) Blood glucose levels measured after overnight fasting (A) or 30 min after i.p. glucose injection (B) in 5-month-old male *Arnt2*^R74C/R74C^ (*n*=7), *Arnt2^R74C/+^* (*n*=8) and *Arnt2^+/+^* (*n*=3) mice. Glucose homeostasis experiments were performed a total of three times with different cohorts of male or female mice. (C) Fasting serum insulin of 5-month-old male *Arnt2*^R74C/R74C^ (*n*=8), *Arnt2^R74C/+^* (*n*=8) and *Arnt2^+/+^* (*n*=4) mice. (D) Liver weights of 4- to 5-month-old male *Arnt2*^R74C/R74C^ (*n*=12), *Arnt2^R74C/+^* (*n*=6) and *Arnt2^+/+^* (*n*=8) mice. (E) Representative hematoxylin and eosin staining of livers from *Arnt2*^R74C/R74C^ and *Arnt2^+/+^* mice. Scale bars: 100 μm. *P<0.05, **P 0.005 and ***P<0.0005 for differences between marked genotypes by one-way ANOVA with *post-hoc* Tukey's test.

The major health consequences of obesity include the development of diabetes and a predisposition towards non-alcoholic fatty liver disease. In order to examine glucose homeostasis in *Arnt2* mutant mice, we measured fasting blood glucose and fasting insulin, and assessed glucose tolerance. Fasted male *Arnt2*^R74C/R74C^ mice displayed elevated circulating insulin and glucose levels compared to fasted wild-type littermate mice (Fig. 3A,C). Thirty minutes after intraperitoneal injection of glucose, male *Arnt2*^R74C/R74C^ mice displayed elevated blood glucose levels, indicating aberrant glucose tolerance (Fig. 3B); a modest heterozygote effect was observed as well. These metabolic effects were also seen in female mice (Fig. S3A-C). These data demonstrate that the *Arnt2*^R74C^ mutation leads to aberrant glucose homeostasis and insulin resistance.

Necropsied *Arnt2*^R74C/R74C^ mice had hepatomegaly (Fig. 3D). Histologically, the *Arnt2*^R74C/R74C^ liver showed evidence of steatosis without obvious inflammation (Fig. 3E). These data demonstrate that the *Arnt2*^R74C/R74C^ mice develop hepatomegaly and non-alcoholic fatty liver disease, presumably as a consequence of obesity and insulin resistance.

### *Arnt2* mutant mice display hyperphagia

**Fig. 4.**
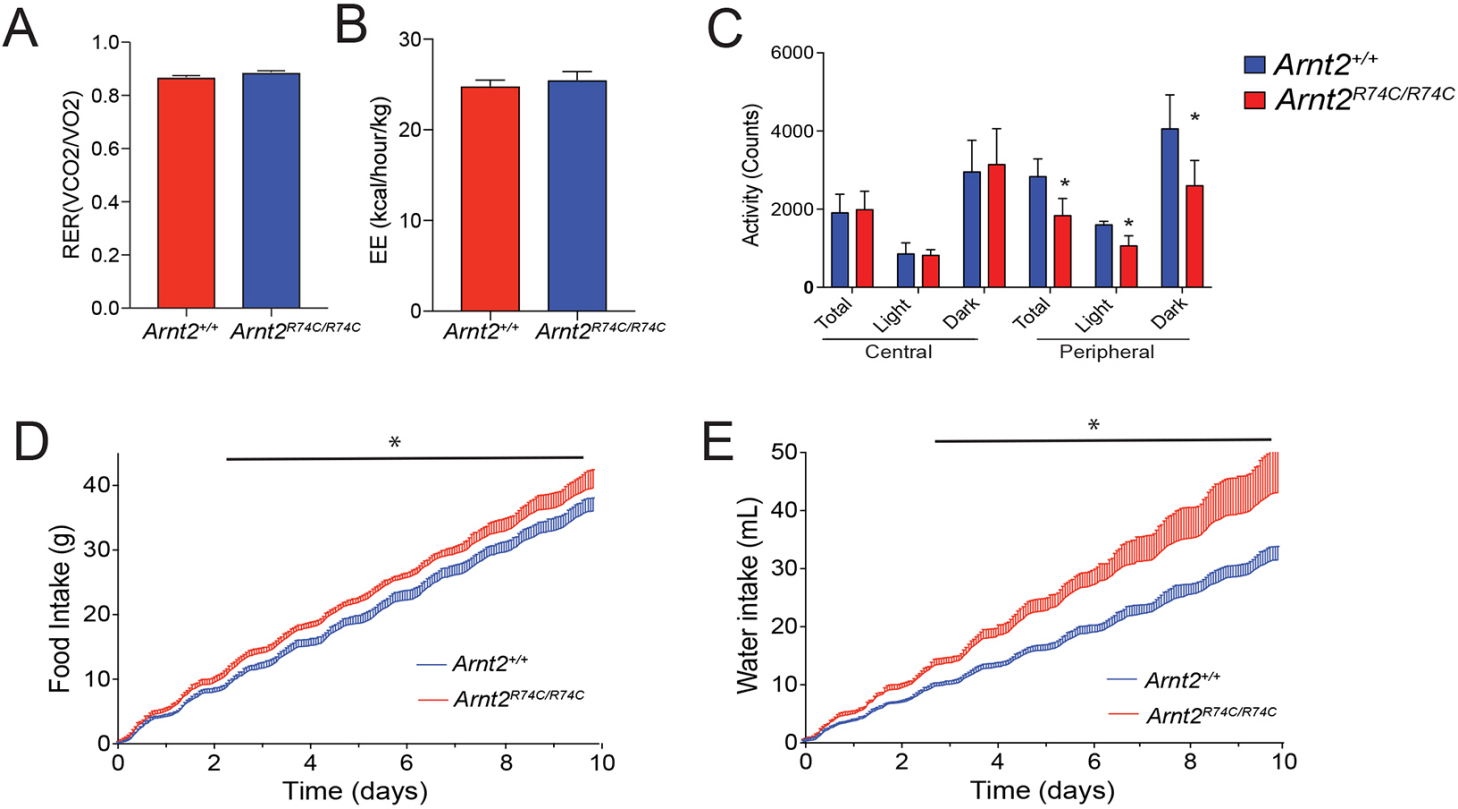
**Metabolic cage analysis of *Arnt2*^R74C/R74C^ mice.** (A) Average respiratory exchange ratio (RER; *V*_CO_2__/*V*_O_2__). (B) Average energy expenditure normalized to lean mass (EE; kcal/hour/kg). (C) Average activity in the peripheral and central parts of the cage. Activity was measured in total as well as in the dark and light phases of the light cycle. For C, *P<0.05 by Student's *t*-test for differences between marked genotype and *Arnt2^+/+^*. (D,E) Cumulative food (D) and water (E) intake of mice during the 10-day study. s.d. shown as bar above line. *P<0.02 by *t*-test with significance adjusted for multiple time points for *Arnt2*^R74C/R74C^ versus *Arnt2^+/+^* for indicated points. For A-E, *n*=6 male *Arnt2*^R74C/R74C^ mice, 6 male *Arnt2^+/+^* mice at 2 months of age. Data are representative of 3 independent experiments.

To better understand the physiological consequences of the *Arnt2* mutation, we examined the metabolic phenotype of the *Arnt2*^R74C/R74C^ mice. After metabolic cage housing for 10 days, male *Arnt2*^R74C/R74C^ mice had energy expenditure and respiratory efficiencies equivalent to those of wild-type mice (Fig. 4A,B). This indicates that *Arnt2*^R74C/R74C^ mice have normal energy expenditures. The *Arnt2*^R74C/R74C^ mice were noted to have a mild decrease in peripheral cage activity, but normal amounts of central cage activity (Fig. 4C). Additionally, the *Arnt2*^R74C/R74C^ mice consumed about 10% more food and 35% more water than wild-type mice (Fig. 4D,E). Taken together, this suggests that the *Arnt2*^R74C/R74C^ obesity phenotype is due to excess caloric intake rather than an overall defect in metabolism.

### *Arnt2* mutant mice display normal hypothalamic cellularity

Compared to wild-type mice, *Arnt2*^−/−^ mice suffer from perinatal lethality and developmental defects of secretory neurons of the hypothalamus (Hosoya et al., 2001). ARNT2 is most highly expressed in the paraventricular nucleus (PVN) as well as the supraoptic nucleus (SON) of the hypothalamus. This expression pattern appears specific and regulated, as no expression is seen in the suprachiasmatic nucleus (SCN) (Michaud et al., 2000). Brains from *Arnt2* mutant mice were grossly identical to those of wild-type mice and histologic analysis did not show any absolute difference in the size of the hypothalamic nuclei nor total neuron density of the PVN, SON and SCN of *Arnt2*^R74C/R74C^ compared to wild-type mice (Fig. S4A,B). These data indicate that hypothalamic development is grossly intact in *Arnt2*^R74C/R74C^ mice and suggest that the defects observed in *Arnt2* mutants likely result from a functional defect in transcription factor activity.

### The *Arnt2*^R74C^ mutation decreases the transcriptional activity of the ARNT2-SIM1 complex

**Fig. 5.**
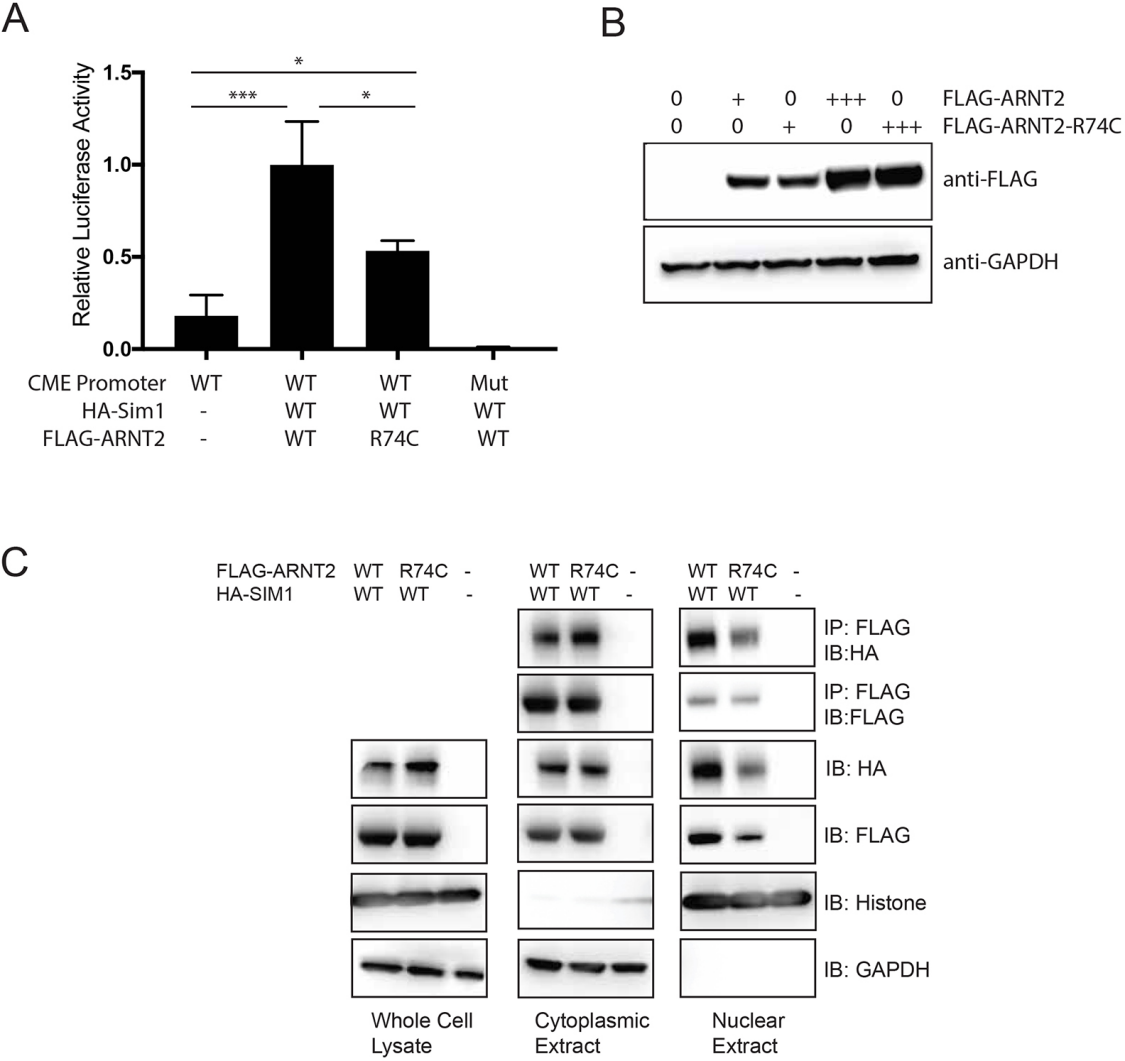
**Decreased transcriptional activity of *Arnt2*^R74C^.** (A) 6×CME-driven luciferase activity in lysates of HEK293T cells transfected with wild-type (WT) *Arnt2* or *Arnt2*^R74C^ (R74C). Results are the average of 3 experiments. *P<0.05 and ***P<0.0005 by one-way ANOVA with *post-hoc* Tukey's test. Mut, mutant CME promoter. (B) Representative western blot of lysates from HEK293T cells transfected with wild-type *Arnt2* or *Arnt2*^R74C^. GAPDH was used as the loading control. (C) Representative immunoprecipitations of HA-SIM1 by FLAG-ARNT2 in cytoplasmic and nuclear fractions of HEK293T cells transfected with *HA-Sim1* and FLAG-tagged wild-type *Arnt2* or *Arnt2*^R74C^. Blots are representative of 4 independent experiments.

bHLH-PAS proteins function as heterodimers, and ARNT2 has been shown to heterodimerize with several members of the family, including NPAS4, SIM1 and SIM2, to form active transcription factors (Bersten et al., 2014, 2013; Jain et al., 1998). SIM1 regulates feeding behavior in both humans and mice (Holder et al., 2000; McMinn et al., 2000; Tolson et al., 2014), and was reported to be the *in vivo* dimerization partner of ARNT2 necessary for development of the hypothalamus (Michaud et al., 2000). The hyperphagic obesity phenotype of *Arnt2*^R74C/R74C^ mice is similar to that observed in SIM1-deficient (*Sim1^+/−^*) mice (Kublaoui et al., 2008). Therefore, we sought to assess the transcriptional consequence of the *Arnt2*^R74C^ mutation in the context of the ARNT2-SIM1 complex. The ability of the complex to activate transcription was assessed using a luciferase reporter gene under the control of six repeats of a bHLH-PAS core binding element [6×CME (CNS midline elements)]. Co-transfection of wild-type ARNT2 with SIM1 in HEK293T cells led to induction of the CME reporter as compared to cells without ARNT2-SIM1 or those with a mutated reporter (Fig. 5A). Cells expressing SIM1 and ARNT2^R74C^ showed a 50% reduction in luciferase activity compared to those expressing SIM1 and wild-type ARNT2 (Fig. 5A). This reduction was not due to protein stability of the mutant since similar levels of protein expression were found upon transfection of FLAG-tagged wild-type ARNT2 and ARNT2^R74C^ in HEK293T cells (Fig. 5B). ARNT2 and ARNT2^R74C^ displayed equivalent heterodimerization to SIM1 in coimmunoprecipitation assays in the cytosol (Fig. 5C). Upon assessing the nuclear fraction of transfected cells, ARNT2^R74C^ itself as well as the ARNT2^R74C^-SIM1 co-precipitating complex were found to be decreased compared to those detected in cells expressing the wild-type ARNT2 protein. This reduced nuclear localization is consistent with another N-terminal mutant of ARNT2, similarly displaying decreased nuclear localization and decreased transcriptional activity (Bersten et al., 2014). These data suggest that *Arnt2*^R74C^ encodes a stable, but hypofunctional, protein due to a failure to translocate to the nucleus.

## DISCUSSION

In the present study, we established a weight-based screen using ENU-mutagenized mice to identify monogenic causes of obesity. Using an isogenic C57BL6/J background, we were able to eliminate variability due to genetic background. Using this unbiased approach, we found a previously undescribed role for the transcription factor ARNT2 in limiting body weight. Our findings indicate that ARNT2 functions to restrict feeding behavior, but has little effect on energy metabolism as measured by respiratory exchange ratio (RER), energy expenditure (EE) and cage activity. As a consequence of hyperphagic obesity, *Arnt2* mutant mice develop diabetes, insulin resistance and hepatic steatosis.

*Arnt2*^R74C^ homozygous mice show similarly increased food consumption to *Sim1^+/−^* mice (10% and 15%, respectively) (Tolson et al., 2010, 2014), which display deficits in neuroendocrine hormones that regulate feeding, such as oxytocin (Kublaoui et al., 2008). Together with evidence that the ARNT2-SIM1 heterodimer is critical for embryonic development of the hypothalamic paraventricular and supraoptic nuclei (Michaud et al., 2000), which control feeding behavior, our findings suggest that ARNT2 cooperates with SIM1 to regulate the function of hypothalamic circuits regulating feeding in adult animals. RNAseq analysis of SIM1-expressing neurons from ARNT2 mutant mice may be informative in identifying ARNT2-SIM1-dependent hormones that regulate feeding. ARNT2 has additional binding partners within the nervous system, including NPAS4 (Bersten et al., 2014); therefore, *Arnt2*^R74C/R74C^ mice may also show CNS transcriptional perturbations distinct from those of SIM1-deficient mice.

Whereas *Arnt2*-knockout homozygotes die perinatally (Hosoya et al., 2001), *Arnt2*^R74C/R74C^ mice are fully viable, suggesting a hypomorphic effect of this missense mutation. Based on a reporter assay for ARNT2-SIM1-dependent transcription *in vitro*, we speculate that ARNT2^R74C^ supports approximately 50% of wild-type levels of transcription, notwithstanding any additional target-specific regulation. This hypomorphic effect appears to probably be due to decreased nuclear translocation, resulting in decreased ARNT2-SIM1 heterodimers in the nucleus. Whatever the level, it is apparently sufficient during embryonic development to permit survival past the perinatal stage, but insufficient in controlling feeding behavior in adults. We note that differences in strain background between the null allele (Hosoya et al., 2001) and the missense allele described here may also contribute to differences in viability of the respective homozygotes.

Forward genetic analysis of ENU-mutagenized mice can, in principle, reveal all non-redundant genes necessary for maintenance of normal body weight. Based on the identification of putatively causative obesity mutations in 15 distinct genes from a survey of 26.5% of mouse genes damaged and tested in the homozygous state three times or more, we estimate that weight-promoting mutations in approximately 56 genes will be identified once 100% saturation is achieved. To date, only *Mc4r* corresponds to a locus found in body mass index genome-wide association studies, a fact which may reflect the relative rarity of viable damaging mutations of the mapped loci in the human population (Locke et al., 2015). Notably, hyperphagia leading to excess caloric intake is a major mechanism of the mapped mutations in this study, such as mutations in *Lep*, *Lepr*, *Alms1*, *Mc4r*, *Ksr2* and *Arnt2*. This is compared to relatively few genes (e.g. *Tub*) with known metabolic or activity phenotypes (Coyle et al., 2008; Stubdal et al., 2000). In the future, applying diet-based interventions such as high-fat or high-carbohydrate diets in forward genetic screening may expose additional obesity genes, and change the underlying etiology of the observed obesity.

## MATERIALS AND METHODS

### Mice

All experiments used mice (*Mus musculus*) of the C57BL/6J strain. Eight- to 10-week-old C57BL/6J mice were purchased from The Jackson Laboratories. ENU mutagenesis of C57BL/6J mice was performed as previously described (Georgel et al., 2008). Mutagenized G0 males were bred to C57BL/6J females, and the resulting G1 males were crossed to C57BL/6J females to produce G2 mice. G2 females were backcrossed to their G1 sires to yield G3 mice, which were screened for phenotypes (Fig. S1). All mice were housed in the University of Texas Southwestern vivarium at an ambient temperature of 21.1-23.1°C. Mice received a standard chow diet containing 16.4% protein and 5% fat (Envigo Teklad 2016). All procedures were approved by the Institutional Animal Care and Use Committee of the University of Texas Southwestern Medical Center and were performed in accordance with institutionally approved protocols.

### Generation of the *Arnt2*^R74C/R74C^ mouse strain using the CRISPR/Cas9 system

To generate the *Arnt2*^R74C/R74C^ mouse strain, female C57BL/6J mice were superovulated by injection of 6.5 U pregnant mare serum gonadotropin (PMSG; Millipore), followed by injection of 6.5 U human chorionic gonadotropin (hCG; Sigma-Aldrich) 48 h later. The superovulated mice were subsequently mated overnight with C57BL/6J male mice. The following day, fertilized eggs were collected from the oviducts and *in vitro*-transcribed *Cas9* mRNA (50 ng/μl) and *Arnt2* small base-pairing guide RNA (50 ng/μl; 5′-ACAGTGAGATTGAGCGGCGC-3′) and single-stranded DNA template (5′-tccttctctttttccctccccctgacctcttgctttctgggaagagagaaccacagtgagattgagcggTGCaggcggaacaagatgactcaatatattacggaactctccgacatggttcccacctgcagtgcactggctag-3′; the mutation site within the template DNA is indicated in upper-case letters) were injected into the cytoplasm or pronucleus of the embryos. The injected embryos were cultured in M16 medium (Sigma-Aldrich) at 37°C in 5% CO_2_. For the production of mutant mice, two-cell-stage embryos were transferred into the ampulla of the oviduct (10-20 embryos per oviduct) of pseudo-pregnant Hsd:ICR (CD-1) female mice (Harlan Laboratories).

### Phenotypic screening, sequencing and determination of candidate genes

Whole-exome sequencing and mapping were performed as described (Wang et al., 2015). Briefly, exome-enriched DNA from all G1 mice was sequenced using the Illumina HiSeq 2500 platform; the G1 mouse of a pedigree carries all possible mutations passed down to its G3 descendants. Prior to phenotypic screening, all G3 mice were genotyped across coding mutations according to their pedigree using Ion Torrent AmpliSeq custom primer panels. Phenotypic screening was performed with the experimenter blinded to genotype and involved weighing individual G3 mice post-weaning; each mouse was weighed one time. To permit comparisons between mice of different ages and genders, body weight change was used instead of raw body weight data. Body weight change was calculated for each mouse relative to the average body weight for a given age and gender, determined from measurements of 10,807 G3 mice on a C57BL/6J background, carrying ENU-induced mutations, and housed under the same conditions (same vivarium, light cycle, feed and water). Body weight change was used as a continuous variable for linkage analysis. Briefly, automated computation of single locus linkage was performed for every mutation in the pedigree using recessive, semi-dominant (additive) and dominant models of transmission using the program Linkage Analyzer (Wang et al., 2015). The magnitude of the quantitative phenotype (here body weight change) was correlated with genotype (REF, homozygous for C57BL/6J reference allele; HET, heterozygous for reference allele and variant allele; or VAR, homozygous for variant allele) at each mutation site in all mice in the pedigree. Continuous variables were analyzed using a linear regression model to assess linkage between specific mutations and quantitative traits. The output of automated mapping was a Manhattan plot of the *P-*value of genotype-phenotype association using recessive, semi-dominant and dominant models of transmission for every mutation in the pedigree.

### Glucose homeostasis testing

Glucose tolerance tests were initiated by intraperitoneal (i.p.) injection of glucose at a dose of 1.5 g/kg body weight after a 16-h fast. Blood was collected from the tail vein for measurement of glucose with the AlphaTRAK glucometer and test strips.

### Histology and microscopy

Freshly isolated livers and brains were fixed in formalin and embedded in paraffin. Hematoxylin-eosin staining was conducted using a standard protocol by the University of Texas Southwestern Histology core. Nissl stain of brains was conducted using a standard protocol by the University of Texas Southwestern Neurohistology core. All brain sections were examined and neuron counting was performed by a neuropathologist, who was blinded to genotypes.

### Metabolic profiling and body composition

Food intake, meal patterns, energy expenditure and locomotor activity were monitored using a combined indirect calorimetry system (Labmaster, TSE Systems GmbH, Germany). Experimental animals were individually housed in a light (12 h on/12 h off, 07:00-19:00)- and temperature (22.5-23.5°C)-controlled environment, and acclimated in the home cage for 5 days before data collection. Mice were then analyzed in the metabolic chambers for 4 days and were provided with food and water *ad libitum*. O_2_ consumption and CO_2_ production were measured by indirect calorimetry to determine energy expenditure. Locomotor activity was measured using a multidimensional infrared light-beam detection system. Continuous food and water intake was recorded using lid-mounted sensors. The diet used in the metabolic cages was 2016 Teklad global 16% protein rodent diets (Envigo, cat. number 2016S). Body composition was assessed by Bruker Minispec mq10.

### Cells and transfection

HEK293T cells (ATCC) were cultured in high-glucose DMEM (Gibco Life Technologies) containing 10% (vol/vol) FBS (HyClone), 10,000 units/ml penicillin and 10,000 μg/ml streptomycin at 37°C. Cells were tested periodically for mycoplasma contamination by Cell Culture Contamination Detection Kit (Thermo Fisher Scientific). Murine *Arnt2* (*mArnt2*) cDNA, *HA-Sim1* cDNA, and 6×CME and mutant 6×CME luciferase were a kind gift from J. Pelletier (Moffett and Pelletier, 2000). *mArnt2* was cloned into a FLAG-tagged expression vector (Clontech). FLAG was immunoprecipitated using magnetic anti-FLAG M2 beads (Sigma). Nuclear and cytosolic lysates were generated using a Cell Fractionation kit (Cell Signaling Technology) as per the manufacturer’s guidelines. The Dual-Glo Luciferase Assay System was performed per assay instructions (Promega). Antibodies for western blot were commercially obtained: anti-FLAG M2 (Sigma, F1804; 1:2000); anti-ARNT2 (Abcam, ab70122; 1:1000); anti-GAPDH (Cell Signaling Technology, 5174S; 1:1000) and anti-histone H3 (Cell Signaling Technology, 9715S; 1:1000).

### Statistical analysis

Age- and sex-matched mice were randomly allocated to experimental groups based on their genotypes. No pre-specified effect size was assumed, and 3-12 mice per genotype were used in experiments; this sample size was sufficient to demonstrate statistically significant differences in comparisons between two or more unpaired experimental groups by unpaired *t*-test or ANOVA, respectively. All mice were included during data analysis. All statistical analyses were performed using GraphPad Prism. Data represent mean±s.d. in all graphs depicting error bars. The statistical significance of differences between experimental groups was determined by Student's *t*-test for comparisons of a single parameter between two groups, or one-way ANOVA with *post-hoc* Tukey's test for comparison of one parameter between multiple groups. All differences with *P*<0.05 were considered significant. *P*-values are denoted by *P≤0.05; **P≤0.005; ***P≤0.0005; ns, not significant with *P*>0.05. Because mice utilized in this study were inbred and age- and sex-matched, variance was assumed to be similar between treatment groups. Phenotypic data were assumed to follow a normal distribution, as has been observed in large datasets from numerous phenotypic screens conducted by our group.

## Supplementary Material

Supplementary information

## References

[DMM035451C1] Adzhubei I. A., Schmidt S., Peshkin L., Ramensky V. E., Gerasimova A., Bork P., Kondrashov A. S. and Sunyaev S. R. (2010). A method and server for predicting damaging missense mutations. *Nat. Methods* 7, 248-249. 10.1038/nmeth0410-248 20354512 PMC2855889

[DMM035451C2] Barsh G. S., Farooqi I. S. and O'Rahilly S. (2000). Genetics of body-weight regulation. *Nature* 404, 644-651. 10.1038/35007519 10766251

[DMM035451C3] Bersten D. C., Sullivan A. E., Peet D. J. and Whitelaw M. L. (2013). bHLH-PAS proteins in cancer. *Nat. Rev. Cancer* 13, 827-841. 10.1038/nrc3621 24263188

[DMM035451C4] Bersten D. C., Bruning J. B., Peet D. J. and Whitelaw M. L. (2014). Human variants in the neuronal basic helix-loop-helix/Per-Arnt-Sim (bHLH/PAS) transcription factor complex NPAS4/ARNT2 disrupt function. *PLoS ONE* 9, e85768 10.1371/journal.pone.0085768 24465693 PMC3894988

[DMM035451C5] Coyle C. A., Strand S. C. and Good D. J. (2008). Reduced activity without hyperphagia contributes to obesity in Tubby mutant mice. *Physiol. Behav.* 95, 168-175. 10.1016/j.physbeh.2008.05.014 18619628 PMC2643381

[DMM035451C6] Georgel P., Du X., Hoebe K. and Beutler B. (2008). ENU mutagenesis in mice. *Methods Mol. Biol.* 415, 1-16. 10.1007/978-1-59745-570-1_1 18370145

[DMM035451C7] Hankinson O. (2008). Why does ARNT2 behave differently from ARNT? *Toxicol. Sci.* 103, 1-3. 10.1093/toxsci/kfn032 18397918 PMC2938178

[DMM035451C8] Hendricks A. E., Bochukova E. G., Marenne G., Keogh J. M., Atanassova N., Bounds R., Wheeler E., Mistry V., Henning E., Korner A. et al. (2017). Rare variant analysis of human and rodent obesity genes in individuals with severe childhood obesity. *Sci. Rep.* 7, 4394 10.1038/s41598-017-03054-8 28663568 PMC5491520

[DMM035451C9] Holder J. L. Jr, Butte N. F. and Zinn A. R. (2000). Profound obesity associated with a balanced translocation that disrupts the SIM1 gene. *Hum. Mol. Genet.* 9, 101-108. 10.1093/hmg/9.1.101 10587584

[DMM035451C10] Hosoya T., Oda Y., Takahashi S., Morita M., Kawauchi S., Ema M., Yamamoto M. and Fujii-Kuriyama Y. (2001). Defective development of secretory neurones in the hypothalamus of Arnt2-knockout mice. *Genes Cells* 6, 361-374. 10.1046/j.1365-2443.2001.00421.x 11318878

[DMM035451C11] Jain S., Maltepe E., Lu M. M., Simon C. and Bradfield C. A. (1998). Expression of ARNT, ARNT2, HIF1 alpha, HIF2 alpha and Ah receptor mRNAs in the developing mouse. *Mech. Dev.* 73, 117-123. 10.1016/S0925-4773(98)00038-0 9545558

[DMM035451C12] Keith B., Adelman D. M. and Simon M. C. (2001). Targeted mutation of the murine arylhydrocarbon receptor nuclear translocator 2 (Arnt2) gene reveals partial redundancy with Arnt. *Proc. Natl. Acad. Sci. USA* 98, 6692-6697. 10.1073/pnas.121494298 11381139 PMC34414

[DMM035451C13] Kleinert M., Clemmensen C., Hofmann S. M., Moore M. C., Renner S., Woods S. C., Huypens P., Beckers J., de Angelis M. H., Schurmann A. et al. (2018). Animal models of obesity and diabetes mellitus. *Nat. Rev. Endocrinol.* 14, 140-162. 10.1038/nrendo.2017.161 29348476

[DMM035451C14] Kublaoui B. M., Gemelli T., Tolson K. P., Wang Y. and Zinn A. R. (2008). Oxytocin deficiency mediates hyperphagic obesity of Sim1 haploinsufficient mice. *Mol. Endocrinol.* 22, 1723-1734. 10.1210/me.2008-0067 18451093 PMC2453606

[DMM035451C15] Locke A. E., Kahali B., Berndt S. I., Justice A. E., Pers T. H., Day F. R., Powell C., Vedantam S., Buchkovich M. L., Yang J. et al. (2015). Genetic studies of body mass index yield new insights for obesity biology. *Nature* 518, 197-206. 10.1038/nature14177 25673413 PMC4382211

[DMM035451C16] McMinn J. E., Baskin D. G. and Schwartz M. W. (2000). Neuroendocrine mechanisms regulating food intake and body weight. *Obes. Rev.* 1, 37-46. 10.1046/j.1467-789x.2000.00007.x 12119644

[DMM035451C17] Michaud J. L., DeRossi C., May N. R., Holdener B. C. and Fan C.-M. (2000). ARNT2 acts as the dimerization partner of SIM1 for the development of the hypothalamus. *Mech. Dev.* 90, 253-261. 10.1016/S0925-4773(99)00328-7 10640708

[DMM035451C18] Moffett P. and Pelletier J. (2000). Different transcriptional properties of mSim-1 and mSim-2. *FEBS Lett.* 466, 80-86. 10.1016/S0014-5793(99)01750-0 10648817

[DMM035451C19] Stubdal H., Lynch C. A., Moriarty A., Fang Q., Chickering T., Deeds J. D., Fairchild-Huntress V., Charlat O., Dunmore J. H., Kleyn P. et al. (2000). Targeted deletion of the tub mouse obesity gene reveals that tubby is a loss-of-function mutation. *Mol. Cell. Biol.* 20, 878-882. 10.1128/MCB.20.3.878-882.2000 10629044 PMC85204

[DMM035451C20] Tolson K. P., Gemelli T., Gautron L., Elmquist J. K., Zinn A. R. and Kublaoui B. M. (2010). Postnatal Sim1 deficiency causes hyperphagic obesity and reduced Mc4r and oxytocin expression. *J. Neurosci.* 30, 3803-3812. 10.1523/JNEUROSCI.5444-09.2010 20220015 PMC3285557

[DMM035451C21] Tolson K. P., Gemelli T., Meyer D., Yazdani U., Kozlitina J. and Zinn A. R. (2014). Inducible neuronal inactivation of Sim1 in adult mice causes hyperphagic obesity. *Endocrinology* 155, 2436-2444. 10.1210/en.2013-2125 24773343 PMC4060186

[DMM035451C22] Turcot V., Lu Y., Highland H. M., Schurmann C., Justice A. E., Fine R. S., Bradfield J. P., Esko T., Giri A., Graff M. et al. (2018). Protein-altering variants associated with body mass index implicate pathways that control energy intake and expenditure in obesity. *Nat. Genet.* 50, 26-41. 10.1038/s41588-017-0011-x 29273807 PMC5945951

[DMM035451C23] van der Klaauw A. A. and Farooqi I. S. (2015). The hunger genes: pathways to obesity. *Cell* 161, 119-132. 10.1016/j.cell.2015.03.008 25815990

[DMM035451C24] Wang T., Zhan X., Bu C.-H., Lyon S., Pratt D., Hildebrand S., Choi J. H., Zhang Z., Zeng M., Wang K.-W. et al. (2015). Real-time resolution of point mutations that cause phenovariance in mice. *Proc. Natl. Acad. Sci. USA* 112, E440-E449. 10.1073/pnas.1423216112 25605905 PMC4321302

[DMM035451C25] Wang T., Bu C. H., Hildebrand S., Jia G., Siggs O. M., Lyon S., Pratt D., Scott L., Russell J., Ludwig S. et al. (2018). Probability of phenotypically detectable protein damage by ENU-induced mutations in the Mutagenetix database. *Nat. Commun.* 9, 441 10.1038/s41467-017-02806-4 29382827 PMC5789985

