# Supplementary material for: A viable hypomorphic Arnt2 mutation causes hyperphagic obesity, diabetes and hepatic steatosis
Source: Dis Model Mech. 2018 Dec 18;11(12):dmm035451. doi: 10.1242/dmm.035451 (PMC6307907; doi:10.1242/dmm.035451)
Supplement: Supplementary information [file dmm-11-035451-s1.pdf]

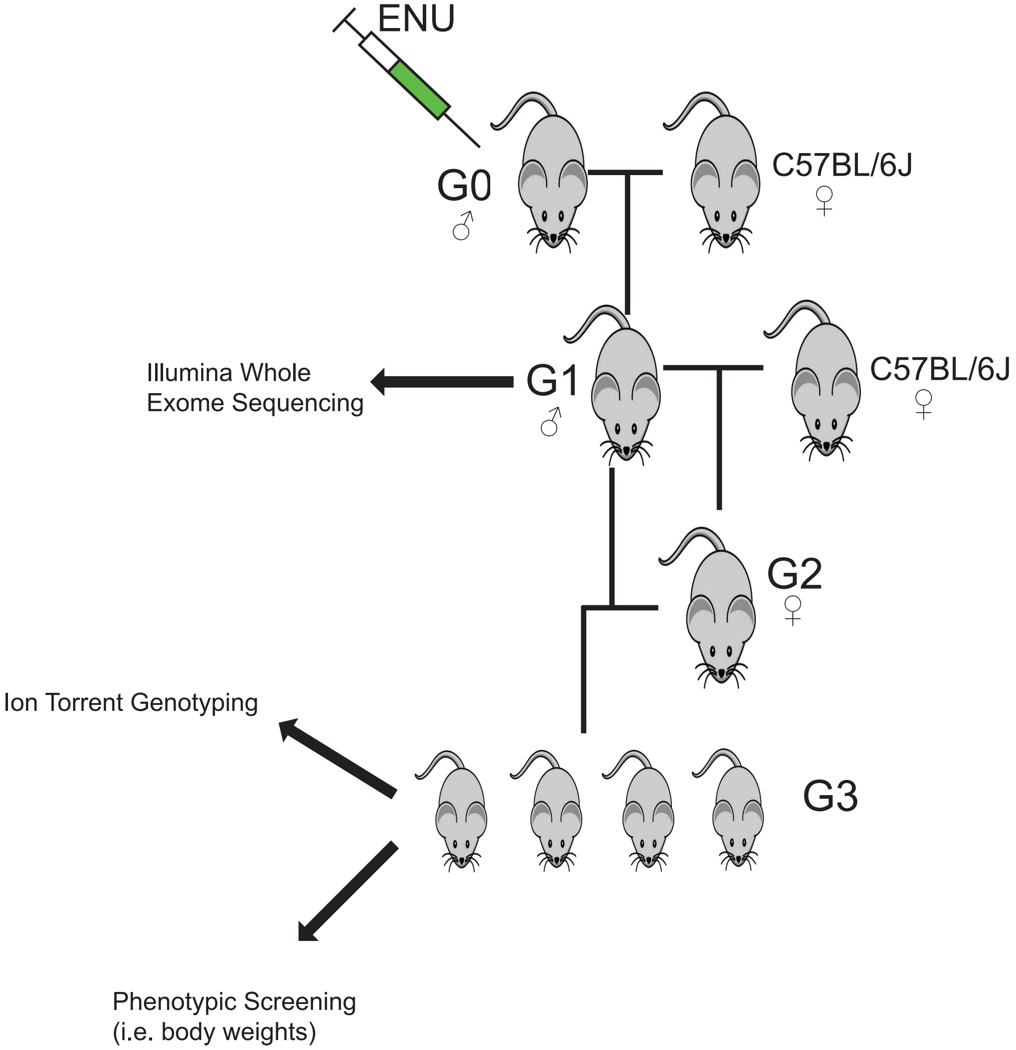

Figure S1: Breeding scheme of ENU mutagenized mice. ENU mutagenized G0 males were bred to C57BL/6J females to generate G1 males. In rare cases, G0 males were bred to female G0' mice, which are female G1s, allowing for greater mutational load and analysis of X-linked traits. All G1 males underwent whole exome sequencing to determine their mutational profile. The resulting G1 males were crossed to C57BL/6J females to produce G2 mice. G2 females were backcrossed to their G1 sires to yield G3 mice, which were screened for phenotypes. All G2 and G3 mice were genotyped according to the mutational profile of the G1 grandsire.

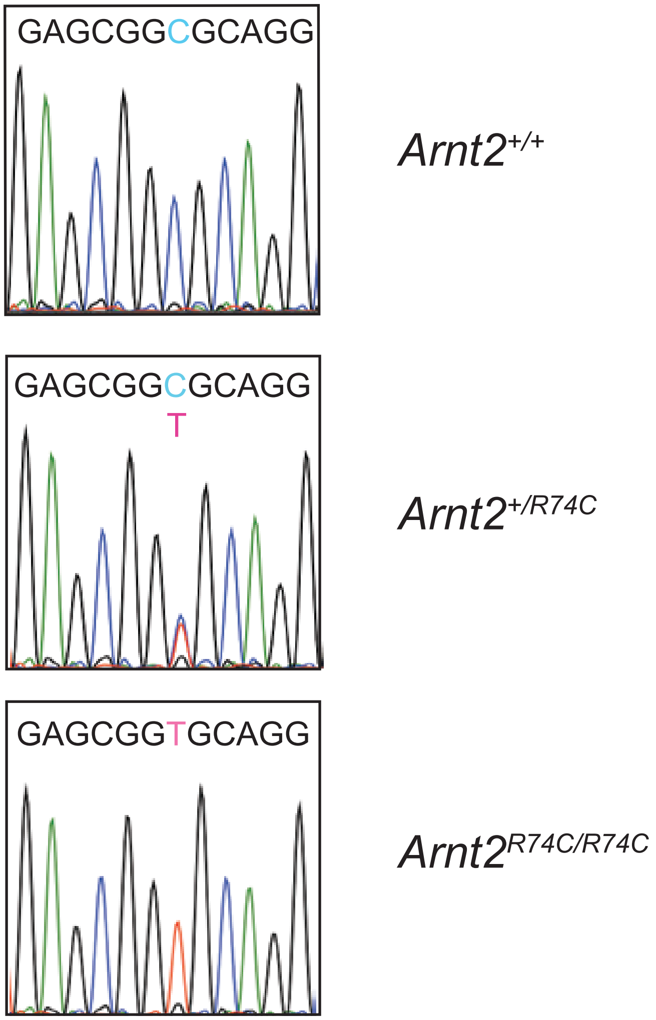

Figure S2: Sequencing chromatogram of DNA from CRISPR/Cas9 targeted mice. Sequenc-ing results of CRISPR/Cas9 targeted mice demonstrating a single nucleotide replacement.

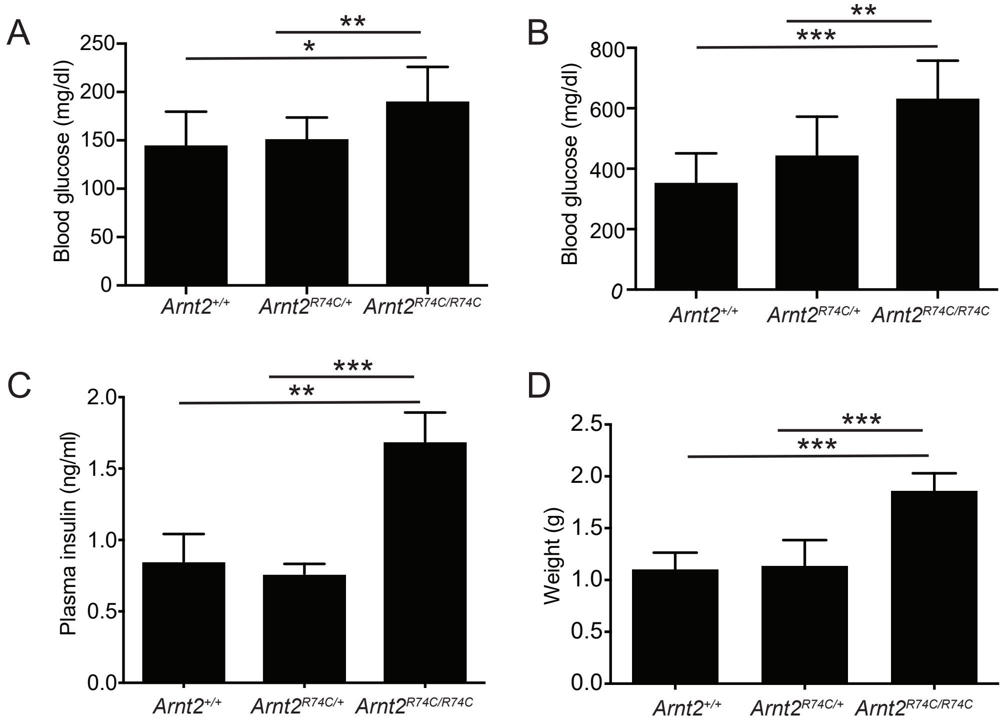

Figure S3: Dysregulated glucose homeostasis in *Arnt2*$^{R74C/R74C}$ mice. (A, B) Blood glucose levels measured after overnight fasting (A) or 30 min after i.p. glucose injection (B) in 5-month-old female *Arnt2*$^{R74C/R74C}$ (n = 8), *Arnt2*$^{R74C/+}$ (n = 16), *Arnt2*$^{+/+}$ (n = 6) mice. Glucose homeostasis experiments were performed a total of three times with different cohorts of mice. (C) Fasting plasma insulin of 5-month-old female *Arnt2*$^{R74C/R74C}$ (n = 10), *Arnt2*$^{R74C/+}$ (n = 17), *Arnt2*$^{+/+}$ (n = 5) mice. (D) Liver weights of 4- to 5-month-old female *Arnt2*$^{R74C/R74C}$ (n = 6), *Arnt2*$^{R74C/+}$ (n = 3), *Arnt2*$^{+/+}$ (n = 4) mice. * $P < 0.05$, ** $P < 0.005$ and *** $P < 0.0005$ for differences between marked genotype and *Arnt2*$^{+/+}$ by one-way ANOVA with post-hoc Tukey's test.

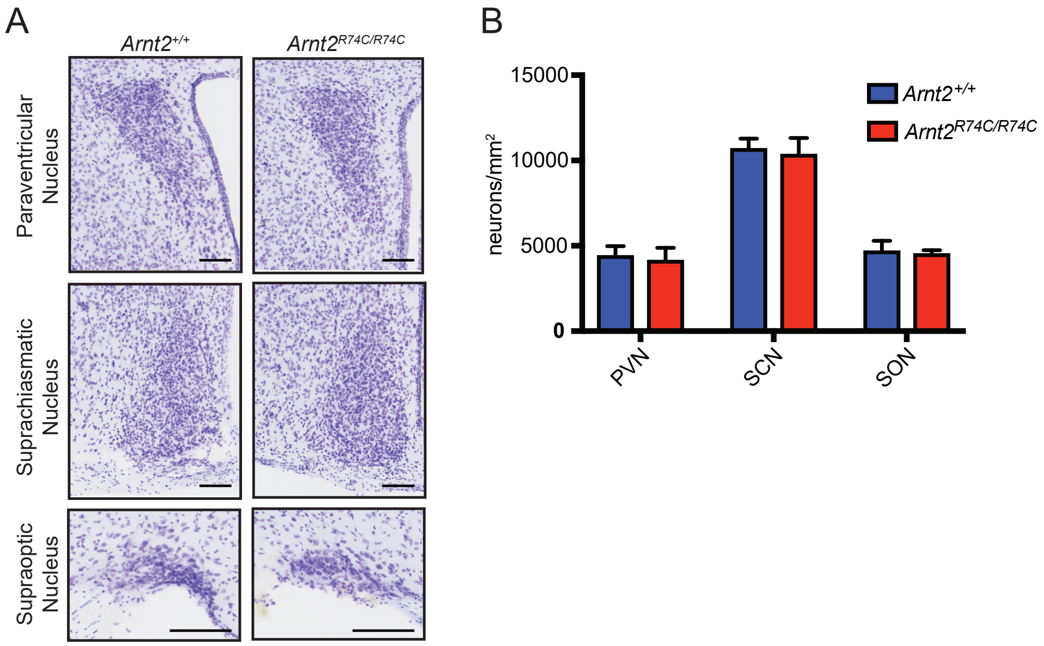

Figure S4: Histology of brain sections from *Arnt2$^{R74C/R74C}$* mice. (A) Representative Nissl stained sections of the paraventricular (PVN), suprachiasmatic (SCN) and supraoptic nuclei (SON) of 6-7-month-old *Arnt2$^{+/+}$* (n=6) and *Arnt2$^{R74C/R74C}$* (n=4) female mice. Scale bar, 100 µm. (B) Quantification of neurons in the PVN, SCN and SON in *Arnt2$^{+/+}$* (n=6) and *Arnt2$^{R74C/R74C}$* (n=4). Experiments were performed one time.
